# Novel Immunoinformatics Approaches to Design Multi-epitope Subunit Vaccine for Malaria by Investigating *Anopheles* Salivary Protein

**DOI:** 10.1038/s41598-018-19456-1

**Published:** 2018-01-18

**Authors:** Rajan Kumar Pandey, Tarun Kumar Bhatt, Vijay Kumar Prajapati

**Affiliations:** 1 Department of Biochemistry, School of Life Sciences, Central University of Rajasthan, Bandarsindri, Kishangarh, Ajmer, 305817 Rajasthan, India; 2 Department of Biotechnology, School of Life Sciences, Central University of Rajasthan, Bandarsindri, Kishangarh, Ajmer, 305817 Rajasthan, India

## Abstract

Malaria fever has been pervasive for quite a while in tropical developing regions causing high morbidity and mortality. The causal organism is a protozoan parasite of genus *Plasmodium* which spreads to the human host by the bite of hitherto infected female *Anopheles* mosquito. In the course of biting, a salivary protein of *Anopheles* helps in blood feeding behavior and having the ability to elicit the host immune response. This study represents a series of immunoinformatics approaches to design multi-epitope subunit vaccine using *Anopheles* mosquito salivary proteins. Designed subunit vaccine was evaluated for its immunogenicity, allergenicity and physiochemical parameters. To enhance the stability of vaccine protein, disulfide engineering was performed in a region of high mobility. Codon adaptation and *in silico* cloning was also performed to ensure the higher expression of designed subunit vaccine in *E. coli* K12 expression system. Finally, molecular docking and simulation study was performed for the vaccine protein and TLR-4 receptor, to determine the binding free energy and complex stability. Moreover, the designed subunit vaccine was found to induce anti-salivary immunity which may have the ability to prevent the entry of *Plasmodium* sporozoites into the human host.

## Introduction

Malaria still remains one of the most devastating and deadly infectious disease, which is characterized by the intermittent high fevers and it’s another form namely cerebral malaria, leads to the neurological complications such as brain injury and coma^1,2^. As per the World Health Organization (WHO) latest estimate, almost 212 million cases of malaria along with 42,900 deaths were reported in December 2016 among 91 countries, worldwide^3^. The high risk of developing malarial infection among a population group depends upon the several factors including the presence of children under the age of 5 years^4^, a patient with HIV co-infection^4^, pregnant woman^4^, mobile population^4,5^, and travelers^6^. The causal organism of malaria is a protozoan parasite belongs to the genus *Plasmodium* and it spreads by the bite of hitherto infected female *Anopheles* mosquito^7^. There are mainly four species of *Plasmodium* namely *P. falciparum*, *P. ovale*, *P. malariae* and *P. vivax* that are responsible for the disastrous diseased condition among human being. Recently, *P. knowlesi* was considered as the fifth species with the ability to cause human malaria infection in Southeast Asian countries mainly Malaysia^8^. If we talk about the severity of infection and distribution of disease, *P. falciparum* is the most severe form of a parasite than other leads to most of the deaths whereas *P. vivax* is the most widely distributed human parasite outside the sub-Saharan region of Africa and cause huge morbidity. The life cycle of malaria parasite consisting of four stages namely liver stage, blood stage, and transmission stage in human being while the last one is mosquito stage and keeping in mind the end goal to destroy the ailment, each stage ought to be considered for treatment^9^. Artemisinin combination therapy is the first-line treatment for *P. falciparum* infection in the region where chloroquine resistance has been evolved. Along with the fast onset of action, artemisinin and its derivatives have rapid clearance from the human body, which needs the combination of the slow-clearing drug to increase the drug efficacy and kills the remaining parasites. The most widely used combination therapies are Artemether-lumefantrine (commercial name: Coartem) and amodiaquine-artesunate (commercial name: Coarsucam)^10^. While the recently approved combination therapies are artesunate-pyronaridine (commercial name: Pyramax) and dihydroartemisinin-piperaquine (Euartesim)^10^. The recent emerging resistance against artemisinin urges to develop some new strategy to prevent the malaria diseases condition^11^. Therefore, in this study, we applied a novel immunoinformatics approach to design multi-epitope based subunit vaccine that may prevent the disease by maintaining the host hemostasis by the inhibition of anticoagulant and anti-inflammatory proteins present in mosquito saliva. It will also inhibit the entry of parasite within the host body by a similar mechanism. Apart from this, if any way parasite enters into the host body, vaccine candidate will stop the salivary protein-mediated induction of parasitic growth.

Among different anopheline vectors, *Anopheles stephensi* is a sub-tropical species that most abundantly present in the Indian subcontinent and also distributed across the Middle East and South Asia region^12^. *A. stephensi* transmit the malarial infection by injecting various *Plasmodium* species to the human host typically via bites. Salivary gland of mosquitos implement various functions for the survival of the vector and complement their feeding behavior by producing a large array of a biochemically active molecule that has immunomodulatory, anti-coagulant and anti-inflammatory properties that disable the host hemostatic response for successful blood feeding^13,14^. Salivary proteins are antigenic and immunogenic in nature which helps the infectivity of parasite^14,15^. D7 protein and salivary apyrase are two different salivary proteins that help in binding, and inhibition of the platelets aggregation, respectively. Salivary peroxidase helps in heme binding and peroxidase activity while Putative TIL domain polypeptide functions as trypsin inhibitor^16^. Recently, it was reported that hamadarin is a 16 kDa protein present in *Anopheles stephensi* saliva which inhibits the activation of plasma contact system^17^ and ultimately blood coagulation. Another protein from *Anopheles gambie* saliva namely gSG6 plays essential blood feeding^18,19^. Recently, Vijay S. *et al*. reported that salivary proteins might be utilized to develop novel antimalarial control strategies via innate immune protection against malaria^16^. These proteins could likewise evoke a host IgG response in natural conditions^20,21^. Mosquito salivary gland surface (SGS) proteins are the prevalent immunogenic component present in saliva having ability to induce immunogenic responses^22^.

This is the reason why we chose the *A. stephensi* salivary proteins from the National Center for Biotechnology Information (NCBI) and subjected to design multi-epitope subunit vaccine. Allergenicity, antigenicity and physiochemical properties were also obtained for the vaccine protein. Moreover, tertiary structure prediction followed by refinement was performed to get a refined 3D model having a higher number of residues in the favored region of Ramachandran plot. Molecular docking and molecular dynamics simulation of vaccine constructs with TLR4 were also performed to check the binding energy and complex stability. Finally, disulfide engineering and *in silico* cloning was performed to increase the stability of vaccine construct and ensuring its effective expression in the microbial system, respectively. This study finally represents a novel approach to develop malaria vaccine using salivary protein instead of parasitic protein, which could be helpful to prevent the *Plasmodium* infection to human host.

## Results and Discussion

### Sequence retrieval of salivary protein and assurance of antigenic conduct

In order to design an immunogenic multi-epitope subunit vaccine, the sum of 33 *A. stephensi* salivary protein sequences was retrieved from the NCBI protein database. Major proteins name is salivary lysozyme, a salivary protein precursor, salivary galectin, salivary lipase, anti-thrombin anopheline, salivary protein SG3, salivary apyrase, salivary secreted serine protease inhibitor, salivary defensin and salivary cecropin. Among 33 salivary protein sequences, only 14 proteins were found to be antigenic as predicted by ANTIGENpro. These 14 sequences were selected based on their score obtained for the probability of antigenicity and all these proteins having a score of ≥08^23^. Obtained score for antigenicity probability clearly denoting the antigenic nature of selected protein sequences which can be used for the subunit vaccine designing^24^.

### CTL epitope prediction and immunogenicity assessment

 Cytotoxic T-lymphocytes are a CD8+ subset of T-cell responses to kill those target cells having intracellular viral, bacterial or protozoan infection^25^. During infection, whenever they encounter to the MHC-I mounted antigen specific to their receptor, they enter the cell cycle and perform several mitotic divisions followed differentiation into the effector cells^26^. Here, we tried to predict the CTL receptor specific immunogenic epitopes using the NetCTL 1.2 server and total 83 CTL epitopes of 9mer length were obtained for the input of 14 salivary protein sequences^27^. In the next step, the immunogenicity of epitopes was determined and as per the instruction of IEDB^28^, higher score indicate greater probability to elicit an immune response; therefore total 21 CTL epitopes with high immunogenicity score were selected and subjected to the vaccine designing (Table 1).

**Table 1:** Predicted cytotoxic T-lymphocyte (CTL) specific epitopes and their immunogenicity score obtained from the immune epitope database.

Serial No.	Accession ID	Epitopes	Comb	IEDB immunogenicity Score	Selected/non selected
1	AAO74839.1	**KLFETTDMY**	0.8412	0.12921	Selected
2	AAO74838.1	**LSDPFDVSV**	1.4235	0.0691	Selected
3	AAO06843.1	**VQGEFKGYY**	0.778	0.04919	Selected
4	AAO06839.1	ALQQGLVDY	0.778	−0.0838	Non-selected
5	AAO06838.1	**HALFWTALY**	1.5459	0.39298	Selected
	AAO06838.1	**LYAEDGLDY**	0.9176	0.17055	Selected
6	AAO06837.1	**FLEDIFSIF**	0.9734	0.23276	Selected
7	AAO06835.1	**TTESTTEAV**	1.3094	0.04773	Selected
	AAO06835.1	**TTSVEDGLI**	0.9071	0.12834	Selected
8	AAO06834.1	**YTHGEEPEY**	1.7764	0.28549	Selected
9	AAO06833.1	**TSDAATTTQ**	1.1906	0.17665	Selected
	AAO06833.1	**WTGPRILPF**	0.918	0.15968	Selected
10	AAO06831.1	**KSERIPVQY**	1.7529	0.1709	Selected
	AAO06831.1	**AQQNEVTEY**	1.0121	0.18351	Selected
11	AAO06829.1	**GLAIEAAPY**	1.1085	0.30749	Selected
	AAO06829.1	**QSWEGHPIY**	0.9192	0.3045	Selected
12	AAO06822.1	**SGDIHSYLY**	1.6689	0.10021	Selected
	AAO06822.1	**SFDNRGNTY**	1.4474	0.00729	Selected
13	AAO06821.1	**IAITQFFGY**	0.7829	0.19826	Selected
	AAO06821.1	**VSSWWSEYL**	0.7536	0.3115	Selected
14	AAL16043.1	**PNDAITHCY**	1.056	0.20851	Selected
	AAL16043.1	**MTLWNAWRL**	0.782	0.467	Selected

### HTL epitope prediction

Helper T-lymphocyte is the key player of both humoral and cell-mediated immune response^29^. Therefore, HTL receptor specific epitopes are probably going to be a crucial part of the prophylactic and immunotherapeutic vaccine^30^. All 14 salivary protein sequences were subjected to IEDB MHC-II epitope prediction module and 8751 epitopes of 15mer length were obtained. In order to become highest immunogenic epitopes, they must have a lower percentile rank and IC_50_ value^24^. Only 14 epitopes with lowest percentile rank ranging from 0.03–0.3 were selected for the vaccine designing (Table 2). Their IC_50_ value ranging from 368–959 denoting that out of 14 epitopes with lowest percentile rank, 7 epitopes have intermediate affinity while remaining to have low affinity for the HTL epitopes. On the other side, all 14 epitopes were found to have IFN-γ inducing capability that was obtained from their positive score on the IFNepitope server output^23,31^ (Supplementary Table 1). All these 14 epitopes were used for the vaccine construction.

**Table 2:** Predicted Helper T-lymphocyte (HTL) specific epitopes and their percentile rank obtained from the immune epitope database.

S. No.	Allele	Epitope	Method	Percentile rank	IC50
1	H2-IAd	VRQEAIARALARAAA	Consensus (smm/nn)	0.03	368
2	H2-IAd	QVRQEAIARALARAA	Consensus (smm/nn)	0.04	415
3	H2-IAd	DQVRQEAIARALARA	Consensus (smm/nn)	0.06	431
4	H2-IAd	RDQVRQEAIARALAR	Consensus (smm/nn)	0.07	430
5	H2-IAd	YRDQVRQEAIARALA	Consensus (smm/nn)	0.09	450
6	H2-IAd	KYYAEMQTTLATVDK	Consensus (smm/nn)	0.11	382
7	H2-IAd	FLAHLLVQASQPWKA	Consensus (smm/nn)	0.17	959
8	H2-IAd	PKYYAEMQTTLATVD	Consensus (smm/nn)	0.18	401
9	H2-IAd	QELRAQIAQQRIAQR	Consensus (smm/nn)	0.18	775
10	H2-IAd	IQELRAQIAQQRIAQ	Consensus (smm/nn)	0.18	716
11	H2-IAd	YYAEMQTTLATVDKA	Consensus (smm/nn)	0.22	616
12	H2-IAd	LAHLLVQASQPWKAL	Consensus (smm/nn)	0.23	946
13	H2-IAd	YAEMQTTLATVDKAK	Consensus (smm/nn)	0.26	747
14	H2-IAd	QYRDQVRQEAIARAL	Consensus (smm/nn)	0.3	754

### Construction of multi-epitope subunit vaccine

A final vaccine construct of 541 amino acid residues was designed using 21 CTL and 14 HTL epitopes as described elsewhere^23,24^ (Supplementary Figure 1). In order to attain maximum immune response TLR-4 agonist (RS09) was used as an adjuvant at the N-terminal site of the vaccine construct^32^. Each joint was occupied by the suitable linkers as described by Nezafat *et al*.^32^, for example, adjuvant and CTL epitopes were combined together by EAAAK linker, intra-CTL and intra-HTL epitopes joint by AAY and GPGPG linker, respectively. Finally, vaccine construct was obtained having adjuvant, linker, CTL, and HTL epitopes in a sequence moving from N-terminal to C-terminal. As this designed subunit vaccine consisting of immunogenic CTL and TTL epitopes along with suitable adjuvant and linker, it may have the ability to inhibit the entry of malaria parasite within the human host body^23,24^.

### B-cell epitope mapping

**Figure 1:**
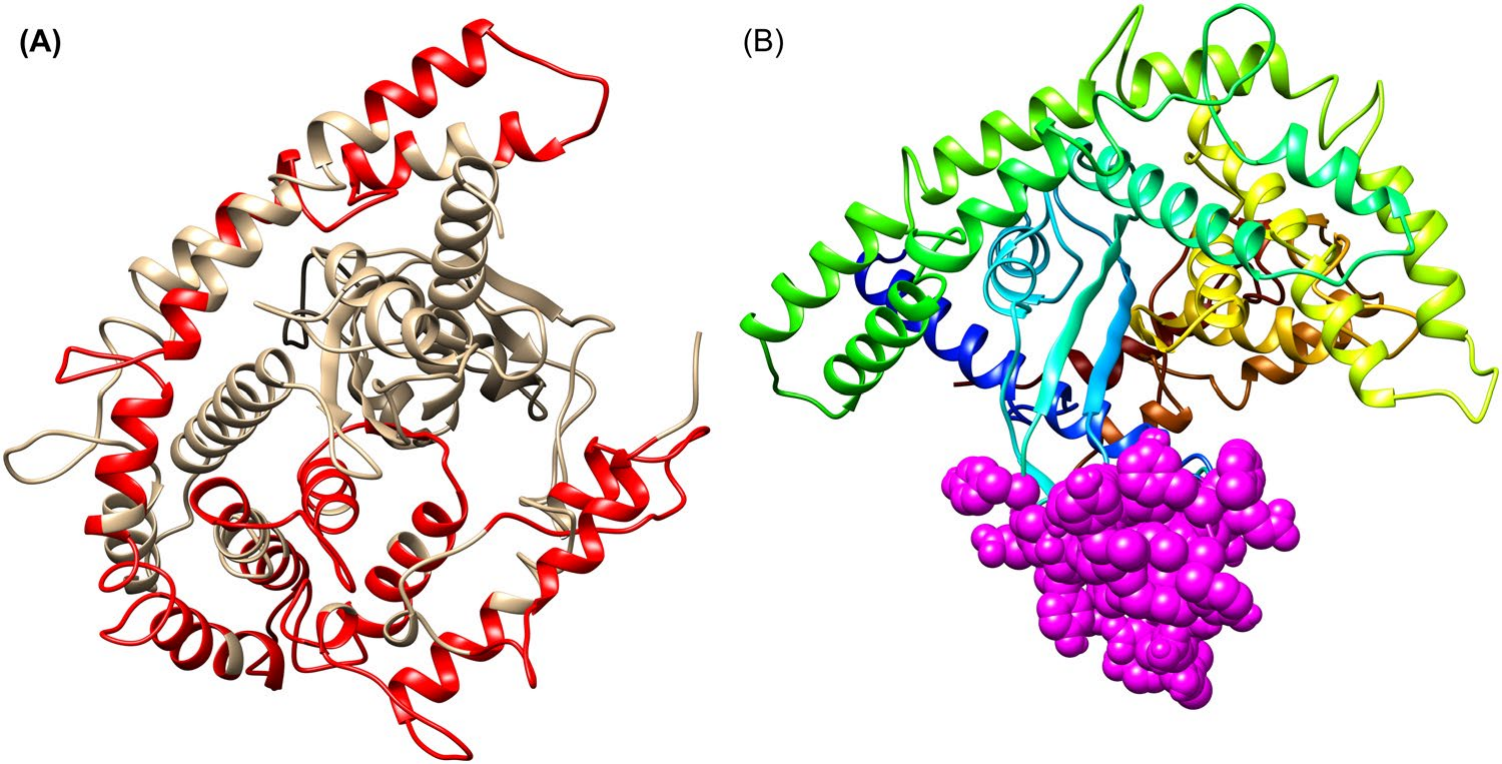
Humoral epitope predictions for subunit vaccine. (**A**) Showing the linear B-cell epitopes (red color) among the 3D structure of final vaccine construct (golden color). (**B**) Conformational B-cell epitopes (magenta color) showing the sequence subunits composed of antigenic epitopes that will come in direct contact with immune receptor.

B-cells are a key player of humoral immunity. An epitope corresponding to the B-cell receptor plays an important role in vaccine design following antibody production^33^. Therefore, BCPREDS server was used to reliably predict the linear B-cell epitopes where BCPRED was the selected prediction method^23^. Total 14 B-cell epitopes of 20mer length were predicted among the primary input sequence of final vaccine construct. Among them, only 11 epitopes were selected and finalized because of their high score of 1.0 (Fig. 1A). Due to the selection of highest scoring B-cell epitope among designed subunit vaccine, our vaccine may have the ability to enhance humoral immunity as well as cell mediated immunity^23^. Discontinuous epitopes of 60 amino acids long were also predicted from the final 3D model of vaccine construct with the probability scoring of 0.791 (Fig. 1B). The obtained probability score also confirming the immunogenic behavior of the designed subunit vaccine^34^.

### Antigenicity and allergenicity prediction of designed vaccine

A vaccine given to human host must be immunogenic in nature and capable to trigger significant humoral immune response which ultimately leads to the memory cell formation against the pathogenic epitopes. The antigenicity of designed vaccine construct was determined by using an alignment-free ANTIGENpro server and found that it has the antigenicity probability of 0.86, which represent the antigenic nature of vaccine construct^24^. The antigenicity score obtained for this vaccine construct is comparable with the antigenicity of subunit vaccine reported elsewhere^24^.

Allergy is an overreaction by our immune system to the previously encountered, ordinarily harmless substance that results in sneezing, wheezing, skin rash, and swelling of the mucous membrane^35^. Allergenicity of predicted vaccine construct was determined using AllerTOP online server and found that the vaccine protein is nonallergic in nature and safe for the human use^23,35^.

### Physiochemical properties assessment

The physiochemical properties of vaccine construct were characterized by using ProtParam server and evaluated for seven parameters. The molecular weight of vaccine protein was found to be 58 kDa which will favor the antigenicity of the vaccine construct^23^. The theoretical pI was found to be 5.61 showing its slightly acidic nature while the total numbers of negative and positive charge residues were 48 and 40, respectively^24^. The estimated half-life in mammalian reticulocytes was 4.4 hours, *in vitro*; while 20 and 10 hours in yeast and *E. coli, in vivo*. The extinction coefficient was found to be 119530 M-1 cm-1, at 280 nm measured in water, assuming that all cysteine residues are reduced. The score obtained for instability index was 29.56, showing the stable nature of vaccine construct. The value of the aliphatic index and Grand average of hydropathicity (GRAVY) was 71.24 and −0.276, respectively. The estimated value of aliphatic index represents the thermostable nature of designed subunit vaccine because higher the value of aliphatic index, greater will be the thermo stability^24^. While, negative value of GRAVY for the input subunit vaccine represents the hydrophilic nature of vaccine^24^. Conclusively, the designed vaccine is immunogenic, thermostable and hydrophilic in nature.

### Tertiary structure prediction, refinement, and validation

**Figure 2:**
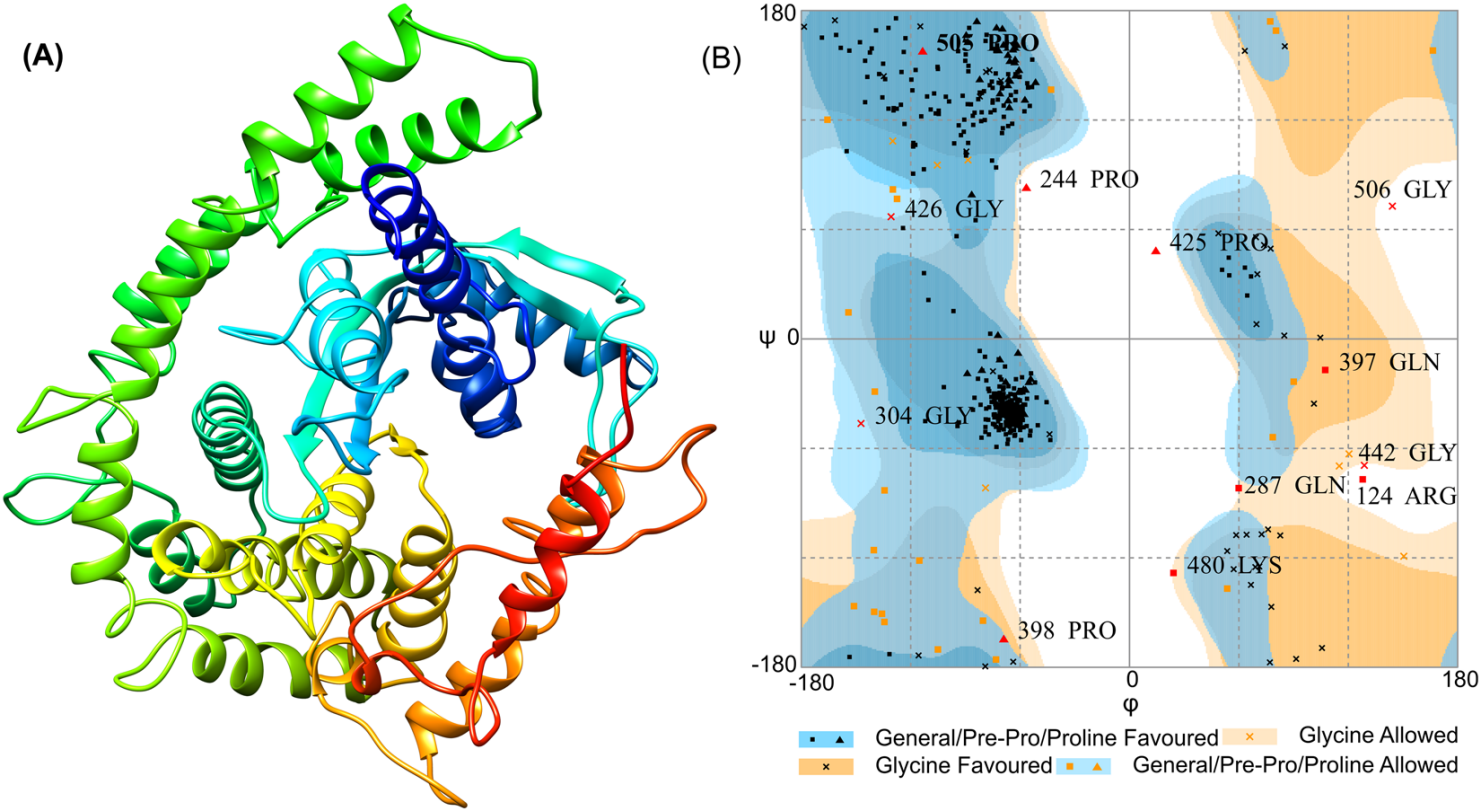
Tertiary structure prediction and validation of vaccine construct. (**A**) Tertiary structure predicted for the primary sequence of subunit vaccine construct showing helix, sheet and coiled region. (**B**) Ramachandran plot formation to validate the 3D modeled structure showing 92.4% residues in the favored region.

The tertiary structure was predicted by using the RaptorX server and 3D model was obtained as described elsewhere^24^ (Fig. 2A). The best template used for the homology modeling was crystal structure of a *Legionella* phosphoinositide phosphatase (PDB ID: 4FYE). Total 541 amino acid residues were modeled as a single domain with 1% disorder. Secondary structure information resulting in the presence of 53% helix, 4% Beta sheet, and 41% coiled structure. P-value is a parameter of homology modeling where low P-value defines the good quality of modeled structure^23^. The P-value obtained for the modeled structure was 6.14e-04 which is low and significant.

Further, protein refinement using GalaxyRefine leads to the increase in a number of residues in the favored region^24^. Initially, 87% of residues were in the Rama-favored region while after refinement the number of residues in the Rama-favored region reached to 92.4%. The refinement output was also validated by plotting Ramachandran plot and found the same that 92.4% residue in Rama-favored region, 5.4% residues in allowed region and only 2.2% residues in outlier region (Fig. 2B).

### Disulfide engineering for vaccine stability

**Figure 3:**
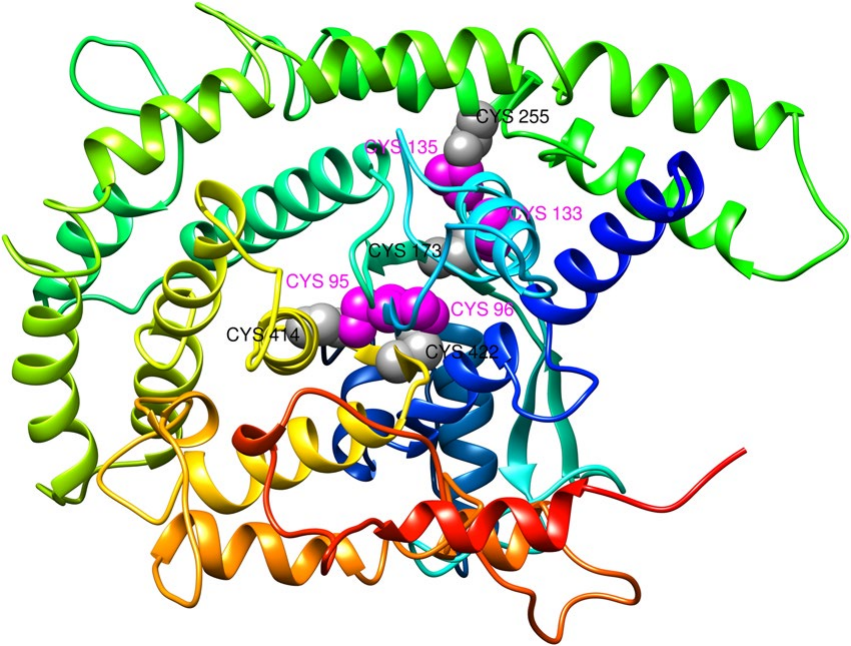
Disulfide engineering to improve protein stability. Showing total 4 mutated residues pairs in magenta and gray color. These residues were selected based on their energy, chi3 value, and B-factor.

In order to stabilize the modeled structure of final vaccine constructs disulfide engineering was performed using Disulfide by design v2.0^36^ and found that there are total 63 pairs of residues that can be used for the purpose of disulfide engineering. But after evaluation on other parameters like energy and Chi3 value, only four pairs of residues were finalized because their value comes under the allowed range i.e. the value of energy should be less than 2.2 and Chi3 should be in between −87 and +97 degree^37^. Therefore, total 8 mutations were created at the residues pairs namely Ala95-Gln414, Tyr96-Gly422, Trp133-Glu173, and Gly135-Phe255 (Fig. 3).

### Codon adaptation and *in silico* cloning

**Figure 4:**
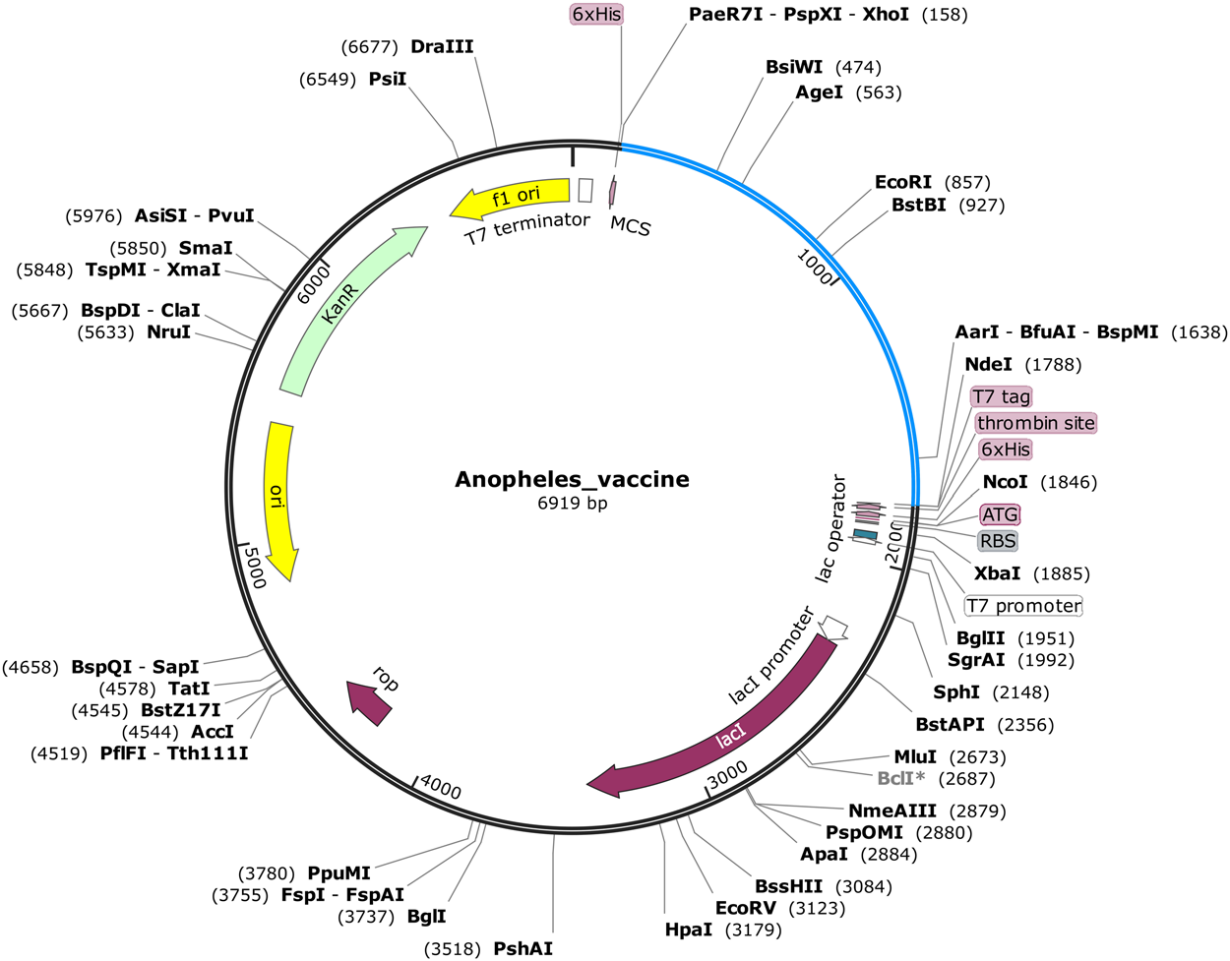
*In silico* cloning for adapted vaccine sequence into pET28a(**+**) vector showing the region of choice in blue color surrounded between XhoI (158) and NdeI (1788) while the vector has shown in black lines.

The main purpose of *in silico* cloning was to express the vaccine protein epitope of *Anopheles* mosquito origin into *E. coli* expression system^23^. Therefore, it was necessary to adapt the codon respective to subunit vaccine construct as per the codon usage of *E. coli* expression system. We adapted the codons as per *E. coli* K12 strain using JCAT server and found that the GC-content of the improved sequence was 58.16% while the value of codon adaptive index was 0.97 which is near to 1.0 that was satisfactory^23^. Later on, XhoI and NdeI restriction sites were created and cloned into the pET28a(+) vector (Fig. 4). The target sequence in the clone is represented in blue color in between aforementioned restriction sites^23^. The target sequence is also enclosed between 6-histidine residues on both ends that will be helpful to the purification purposes. The total length of the clone was 6.9kbp.

### Molecular docking of vaccine constructs with TLR4

**Figure 5:**
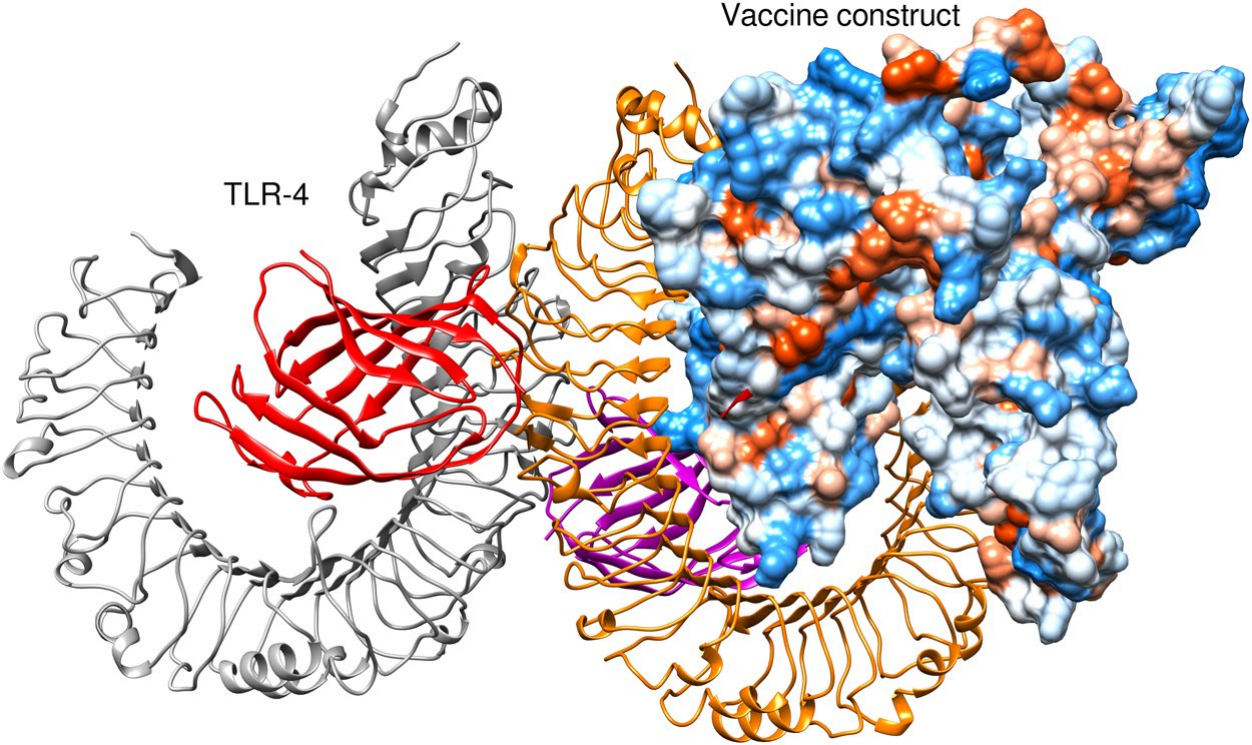
Docked complex of vaccine protein and TLR-4 receptor here vaccine construct is shown in the sphere form, docked within the TLR-4 receptor

Molecular docking of subunit vaccine protein and TLR-4 receptor was performed using the ClusPro 2.0 and total 30 models were generated^38^. Among them, only that model was selected which properly occupied the receptor and having lowest energy score and found that model number 0.00 fulfill the desired criteria that’s why selected as the best-docked complex (Fig. 5). The energy score obtained for the model 0.00 was found to be −1187 which is lowest among all other predicted docked complex showing highest binding affinity.

### Molecular dynamics simulation for Vaccine-TLR4 complex

**Figure 6:**
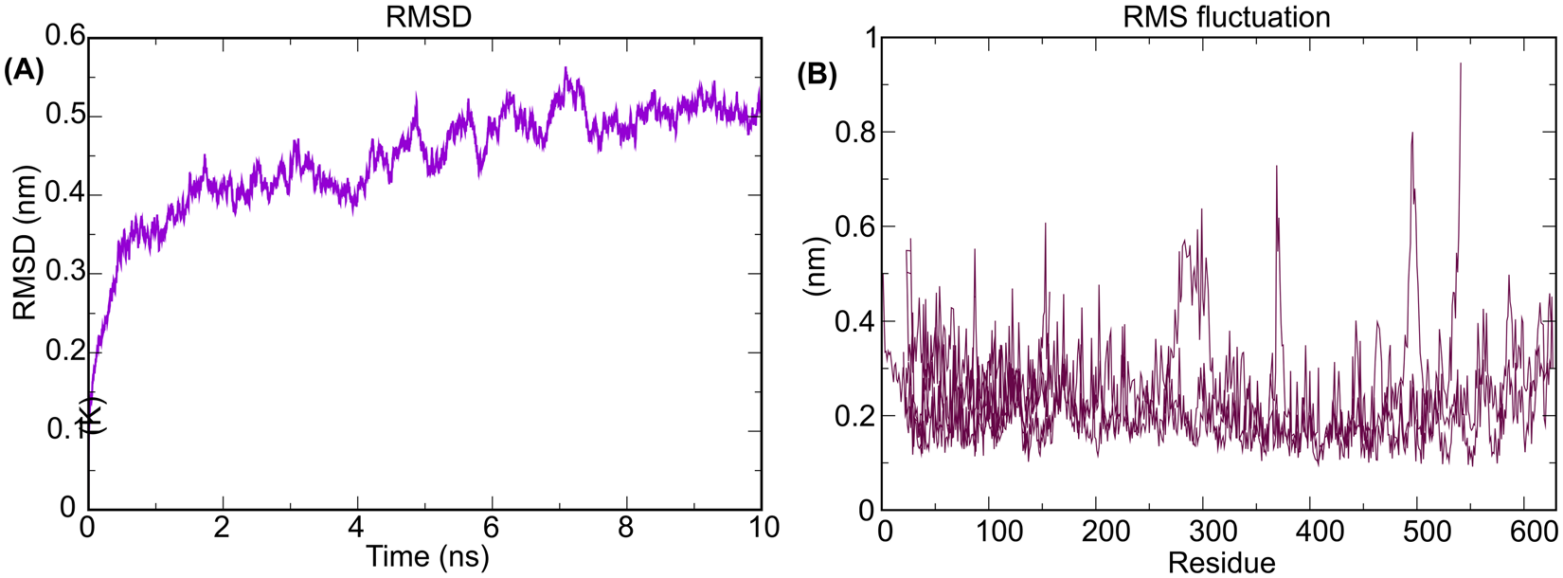
Molecular dynamics simulation output of vaccine protein-TLR-4 docked complex. (**A**) RMSD obtained for the complex backbone showing that initially, RMSD increased up to 8 nanoseconds after that become stable at 0.5 nanometers, while (**B**) showing an average RMSF of 0.2 for the side chain residues of the docked complex.

Molecular dynamics simulation was performed using Gromacs 5.1.5 using a GROMOS9643a1 force field^39^. The potential energy obtained for the complex was −9.9 KJ/mol, while the value of temperature, pressure, and density was obtained as 299.77 K, 2.54 bar and 1016.4 kg/m^3^ (Supplementary Figure 2A–C). The radius of gyration obtained for the docked complex showing that the distance in rotating complex from the center of mass is 4.3 nanometers that decreases up to 4.25nanometers at the time duration of 10 nanoseconds (supplementary Figure 2D). The RMSD value of protein backbone was 0.4 nanometers (Fig. 6A) while RMSF score obtained for the protein side chain was found to be 0.2 nanometers (Fig. 6B). Both these scores are satisfactory showing strong complex stability^23,24^.

## Conclusion

Malaria is severe infection characterized by the high fever with irregularity but may also lead to brain injury and coma. It affects 212 million people from 91 countries, worldwide. Lack of effective vaccine and emergence of resistance against artemisinin created a disastrous condition among the people living in the endemic zone. Therefore, it’s the need of time to search for the new options to tackle this severe problem. The vector for malaria is the *Anopheles stephensi* in the Indian subcontinent leads to the transfer of malaria parasite. Literature survey reveals that salivary proteins of *Anopheles* mosquito not only supports the pathogenesis but are also immunogenic in nature. Therefore, this study was designed to reach a step ahead in the path of vaccine development. We used the primary amino acid sequence of *Anopheles* mosquito salivary protein to design a subunit vaccine construct. The constructed vaccine has CTL, HTL and BCL epitopes of varying length. It has antigenic properties in the absence of allergenic properties. It was stable and having a good binding affinity for the TLR-4 receptor. Collectively, this study applied a series of immunoinformatics tools in a sequential manner to find an effective vaccine that may fight against the malaria infection. However, this study needs experimental validation to prove this computational work. The experimental work may include the synthesis of designed subunit vaccine followed by the *in vitro* and *in vivo* analysis to determine the immunogenicity and safety concern of the same.

## Methodology

### *Anopheles stephensi* salivary protein sequence retrieval and assurance of antigenic conduct

*Anopheles stephensi* mosquito is the vector of malaria transmission to the human being in the Indian subcontinent. While the salivary gland proteins of *Anopheles* mosquito was reported for their role in parasite pathogenesis and having the ability to induce IgG response in the natural host^20,21^. Therefore, total 33 salivary proteins of *A. stephensi* were obtained from the national center for biotechnology information (NCBI) protein database (Retrieval date 25/08/2017) and subjected to multi-epitope vaccine designing. As the main purpose of vaccination is to induce an immunogenic response within the host body, all the retrieved protein sequences were subjected to their antigenicity prediction using ANTIGENpro. Based on the antigenicity result, only 14 proteins were found to have an antigenic probability of ≥0.8 were selected and used in the next step.

### Cytotoxic T-lymphocyte (CTL) epitope prediction and immunogenicity assessment

CD8+ cytotoxic T-lymphocyte were shown to inhibit malaria parasitic growth and development, inside the hepatocytes cells^40^. To get an immunogenic CTL epitopes having the ability to elicit cell-mediated immunity and form the memory cells, all 14 salivary protein sequences were subjected to the NetCTL 1.2 server^41^. NetCTL 1.2 is an online web server intended for predicting CTL epitopes among input protein sequences based on the training dataset. NetCTL was selected to predict the CTL epitopes because of its higher prescient execution on all execution parameters as compared to the recently developed servers namely MHC-pathway, MAPPP, and EpiJen. All the salivary protein sequences were submitted in the FASTA format to predict the CTL epitope at the threshold score of 0.75 (default). Those epitopes having a combined score of greater than 0.75 were selected as CTL epitope and further subjected to the Immune Epitope Database (IEDB) MHC class I immunogenicity prediction module. Predicted CTL epitopes for each salivary protein was used as an input sequence and the result was obtained in the form of the score, where higher score determines that greater will be the probability of eliciting an immune response.

### Helper T-lymphocyte (HTL) epitope prediction

Helper T- cell response is the major part of cell-mediated immunity and helps in pathogen clearance by the help of various cytokines and immune cells^42,43^. They have the ability to induce both CTL and humoral immune response by the secretion of lymphokines like IL-2, IL-4, IL6, Granulocyte-macrophage colony-stimulating factor (GM-CSF), and IFNγ. In view of that, we can say that HTL epitopes mainly of Th1 type are most likely going to be a crucial part of the prophylactic and immunotherapeutic vaccine. Therefore, IEDB MHC-II epitope prediction module was used to predict the HTL epitopes for all 14 *Anopheles* salivary protein sequences^44^. The available parameters were kept default except for allele selection where the nominated alleles were H2-IAb, H2-IAd, and H2-IEd. Output epitopes were ranked based on their percentile rank score where lower percentile rank representing that greater will be the binding affinity for HTL receptor. Secondly, to prove our work that the predicted HTL epitopes will have ability to activate Th1 type immune response followed by the IFN-γ production, top 14 predicted HTL epitopes were subjected to the IFN epitope server using predict option. All 14 epitopes were submitted in the FASTA format followed by the approach selection and model of prediction. Motif and SVM hybrid was selected as the approach and IFN-gamma versus other cytokine as model of prediction.

### Designing of multi-epitope subunit vaccine

So as to plan an appropriate vaccine candidate, it must have the ability to induce CTL and HTL immune response. In other words subunit vaccine must contain both CTL and HTL epitopes along with suitable linkers. Keeping in mind the end goal to effectively activate both innate and adaptive immune response, subunit vaccine must consist of a strong immunostimulatory adjuvant. In the previous decades, there is a huge headway in the adjuvant engineering, for instance, Toll-like receptor (TLR) agonists have made its contribution as a part of peptide-based subunit vaccine as a functional option for present-day immunotherapy^45^. Recently, Junqueira *et al*. have shown that CpGs oligodeoxynucleotides (CpG ODNs) and Glycoinositolphospholipids (GIPL) gotten from *Trypanosome cruzi* having the ability to activate TLR-4 and TLR9 leads to actuate potent pro-inflammatory reaction^46^. Secondly, proteo-glycolipid complex (P8GLC) derived from *Leishmania* parasite has shown its affinity for the TLR-4 receptor and recognized as ligand^47^. Moreover, TLRs having the capability to recognize the *Plasmodium* ligands, for example, *Plasmodium falciparum* primes the human TLR-4 response towards high proinflammatory cytokine profile^48^. Shanmugam A. *et at*. has reported that synthetic TLR-4 agonist namely RS-09 (Sequence: APPHALS) can be used as a novel class of adjuvant^49^, therefore, it was added as an adjuvant and linked with epitopes (CTL and HTL) by using EAAAK linker^50^. Linkers assume an imperative part in simulating the vaccine construct to work as an independent immunogen and producing higher antibody titer than that of single immunogen^51^. Total three linkers namely EAAAK, AAY, and GPGPG, were used to construct the final vaccine. AAY and GPGPG linkers were added at the intra-epitope position to link the CTL and HTL epitopes, respectively.

### B-cell epitope prediction

B lymphocytes, a type of white blood cells, are the key player of humoral immunity by antibody production. The identification of B-cell epitopes is an essential part in vaccine designing. BCPREDS server was used to predict the linear B-cell epitopes of 20 amino acids long. The amino acid sequence of final vaccine construct was used as an input sequence in plain format followed by the selection of fixed length epitope prediction method and length of the epitope. BCPREDS (default method) was selected as the prediction method for the epitope of 20 amino acids long^52^. The specificity threshold was set to be by default at 75% to get the result in a user-friendly format. While conformational epitopes were predicted using ElliPro server for the input of tertiary protein structure of vaccine construct.

### Antigenicity and allergenicity prediction of designed vaccine

Antigenicity determines the ability of an antigen to binds with the B- and T-cell receptor that may lead to the immune response and memory cell formation. Therefore, the antigenic nature of predicted vaccine construct was determined to ensure its ability to interact with the immune receptor. ANTIGENpro is a sequence based, pathogen independent and alignment-free prediction method that was used to check the antigenic behavior of vaccine protein. It uses SVM classifier to summarize the probable antigenic or non-antigenic nature of proteins. ANTIGENpro uses the existing protein antigenicity microarray files of eight feature sets for five pathogens to construct two-stage architecture; among them, the first one is multiple representations of the primary protein sequence and the second one is five machine learning algorithms.

Allergenicity is the potential of a material to cause sensitization and allergic reactions associated with the IgE antibody response. Therefore, the predicted vaccine construct must be free from the allergenic nature. AllerTOP v. 2.0 was used to check the allergenicity of the vaccine construct based on the method that uses auto cross-covariance (ACC) transformation of protein sequences into uniform equal-length vectors. Input protein sequence of vaccine protein was classified by the k-nearest neighbor algorithm (kNN, k = 1) which is based on the training set of 2427 known allergen from different species and 2427 nonallergen from similar species.

### Physiochemical properties assessment

The main purpose of vaccination is to induce an immune response after injecting the vaccine into the body. Therefore, it is necessary to define the physical and chemical parameters associated with the vaccine. ProtParam^53^ web server, a part of Expert Protein Analysis System (EXPASY), was used to define various physicochemical properties of predicted vaccine construct. The primary protein sequence of the vaccine was used to predict the various parameters including molecular weight (kDa), estimated half-life, theoretical pI, aliphatic index, grand average of hydropathy (GRAVY) and so on.

### Tertiary structure prediction

Protein molecule achieves maximum stability in its lowest energy state by proper bending and twisting to form a tertiary structure. It is the interaction between the amino acids side chain residue which is responsible to stabilize the protein structure. The 3-dimensional structure of predicted vaccine construct was obtained by utilizing RaptorX structure prediction server. RaptorX is a pure *ab initio* method that can be used to build a 3D model in a template-free manner.

### Refinement of 3D vaccine model and validation

It is the degree of likeness between the target and available template structure that determines the quality of protein model structure created by contemporary protein structure prediction techniques^39,54^. Therefore, it was necessary to improve the template based predicted model beyond the accuracy by utilizing the template information. To fulfill this thought, output model of RaptorX server was subjected to the GalaxyRefine web server^55^, which is based on the CASP10 tested refinement method. GalaxyRefine performs rehashed structure perturbation followed by overall structural relaxation by performing molecular dynamics simulation.

### Disulfide engineering for vaccine stability

Before proceeding to the next step, it was necessary to improve the stability of refined protein model. Disulfide bonds are covalent interactions that emulate the stabilizing molecular interaction and provide a considerable stability to protein model by confirming precise geometric conformations. Disulfide engineering is a novel approach for creating disulfide bonds into the target protein structure. Therefore, the refined model of final vaccine construct was subjected to the Disulfide by Design 2.0^36^ to perform disulfide engineering. Initially, the refined protein model was uploaded and run for the residue pair search that can be used for the disulfide engineering purpose. Total 4 residue pairs were selected to mutate them with cysteine residue using create mutate function of the Disulfide by Design 2.0 server.

### Codon adaptation and *In silico* cloning

Codon adaptation is a way to attain major expression rate of foreign genes in the host when the codon usage of the host differs from that of the organism where the gene stems from. Unadapted codon may lead to the minor expression rate in the host. Therefore, the primary sequence of vaccine protein was submitted to the Java Codon Adaptation Tool (JCAT) to adapt their codon usage to most sequenced prokaryotic organisms (*E. coli* K12)^23^. CAI value and GC content of the adapted sequence was also obtained. Later on, the adapted nucleotide sequence corresponding to the designed vaccine construct was cloned into the *E. coli* pET28a(+) vector by using the restriction cloning module of SnapGene tool^24^.

### Molecular docking of vaccine constructs with TLR4

Molecular docking is a computational method used to predict the preferred orientation of ligand molecule to the receptor molecule in their stable complex form^56^. It can be also used to predict the binding affinity between these two molecules in terms of scoring function. As mentioned in the previous section, TLRs having the capability to recognize the *Plasmodium* ligands and *P. falciparum* primes the human TLR-4 response towards high proinflammatory cytokine profile^48^. Therefore, TLR-4 was selected as receptor and its PDB file (PDB id: 4G8A) was obtained from RCSB-Protein Data Bank while the refined model of vaccine protein was used as a ligand. Protein-protein docking was performed using the ClusPro 2.0: protein-protein docking server, to check the binding affinity between them^57^.

### Molecular dynamics simulation for Vaccine-TLR4 complex

Molecular dynamics simulation is a widely accepted computational approach which is used to determine the stability of protein-ligand complex at the microscopic level^58^. The protein-protein docked complex output of ClusPro was used as an input to perform the molecular dynamics simulation using Gromacs v5.1.5. Initially, the crystal water of complex was removed followed by the topology generation using a GROMOS9643a1 force field. In the next step, protein complex was centered in a cubic boundary box and filled by water molecule using simple point charge (SPC) water model and chloride ion was used for the charge neutralization of complex. Moreover, energy minimization followed by canonical equilibration (NVT ensemble) and isothermal-isobaric (NPT ensemble) was performed for a time duration of 100 ps. Finally, molecular dynamics simulation was executed for the time duration of 10ns^59^. The root mean square deviation (RMDS) for backbone and root mean square fluctuation (RMSF) for side chain was determined.

## Electronic supplementary material

Supplementary Info
