# Supplementary material for: Novel Immunoinformatics Approaches to Design Multi-epitope Subunit Vaccine for Malaria by Investigating Anopheles Salivary Protein
Source: Sci Rep. 2018 Jan 18;8:1125. doi: 10.1038/s41598-018-19456-1 (PMC5773588; doi:10.1038/s41598-018-19456-1)
Supplement: Supplementary file 1 — Supplementary Info [file 41598_2018_19456_MOESM1_ESM.pdf]

Novel Immunoinformatics Approaches to Design Multi-epitope Subunit Vaccine for Malaria by Investigating *Anopheles* Salivary Protein

Rajan Kumar Pandey[1], Tarun Kumar Bhatt[2], Vijay Kumar Prajapati[1]*

[1]Department of Biochemistry, School of Life Sciences, Central University of Rajasthan, Bandarsindri, Kishangarh, Ajmer 305817, Rajasthan India.

[2]Department of Biotechnology, School of Life Sciences, Central University of Rajasthan, Bandarsindri, Kishangarh, Ajmer 305817, Rajasthan India.

*Corresponding author
Prof. Vijay Kumar Prajapati,
Department of Biochemistry
Central University of Rajasthan
NH-8, Bandarsindri, Ajmer
Rajasthan Pin- 305817 India
Phone: +91 7597271362
Email: vkprajapati@curaj.ac.in

Supplementary figure 1: Primary sequence of final subunit vaccine constructs

APPHALSEAAAKMTLWNAWRLAAYPNDAITHCYAAYVSSWWSEYLAAYIAITQFFGYAAYSFDNRGNTYAAYSGDIHS
YLYAAYQSWEGHPIYAAYGLAIEAAPYAAYAQQNEVTEYAAYKSERIPVQYAAYWTGPRILPFAAYTSDAATTTQAAYY
THGEEPEYAAYTTSVEDGLIAAYTTESTTEAVAAYFLEDIFSIFAAYLYAEDGLDYAAYHALFWTALYAAYVQGEFKGYYA
AYLSDPFDVSVAAYKLFETTDMYGPGPGVRQEAIARALARAAAGPGPGQVRQEAIARALARAAGPGPGDQVRQEAIA
RALARAGPGPGRDQVRQEAIARALARGPGPGYRDQVRQEAIARALAGPGPGKYYAEMQTTLATVDKGPGPGFLAHLL
VQASQPWKAGPGPGPKYYAEMQTTLATVDGPGPGQELRAQIAQQRIAQRGPGPGIQELRAQIAQQRIAQGPGPGYY
AEMQTTLATVDKAGPGPGLAHLLVQASQPWKALGPGPGYAEMQTTLATVDKAKGPGPGQYRDQVRQEAIARAL

APPHALS= Adjuvant

EAAAK, AAY & GPGPG= Linkers

9mer epitope= CTL epitope

15mer epitope= HTL epitope

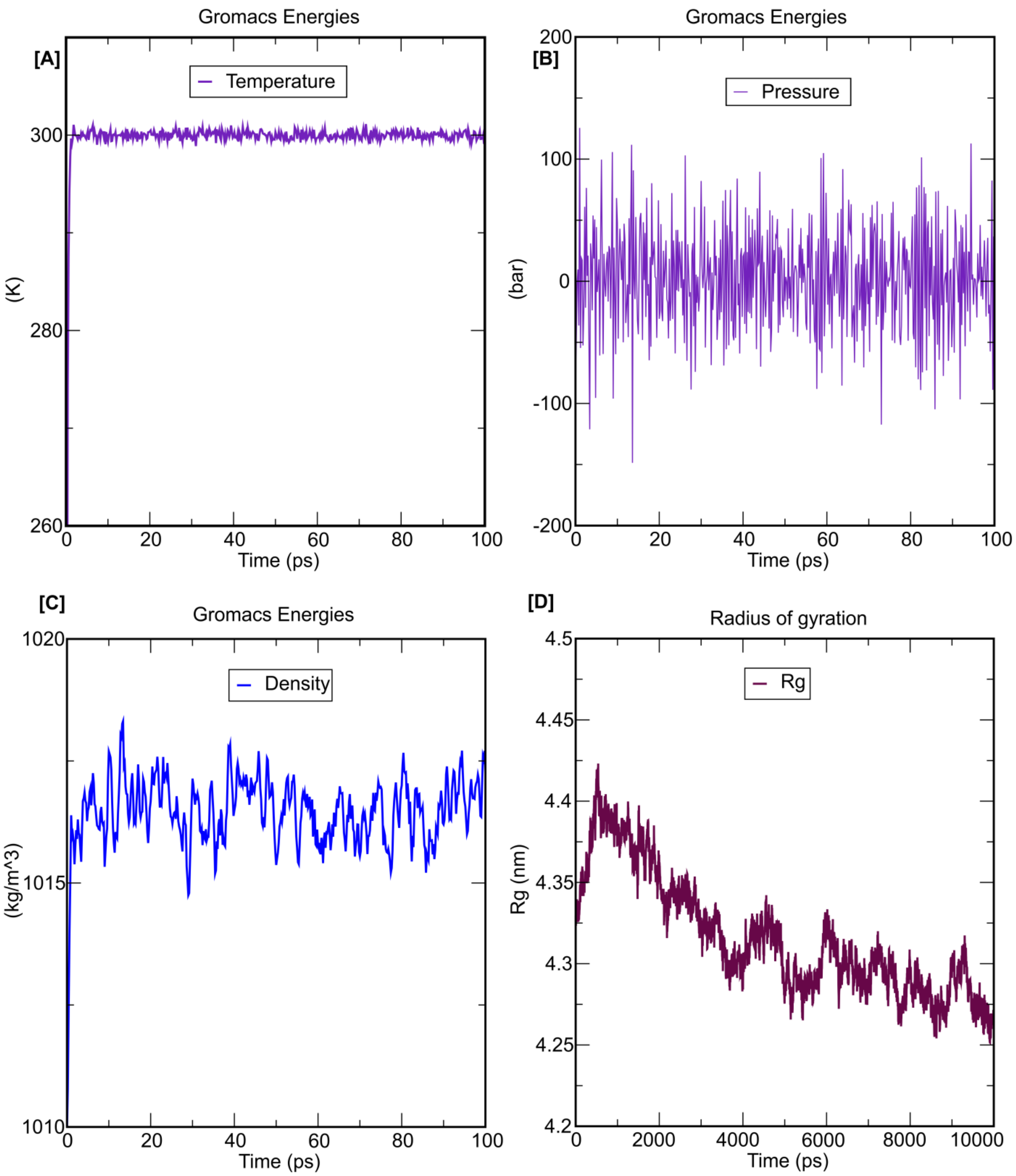

Supplementary figure 2: Molecular dynamics simulation output showing **(A-C)** Gromacs energy of temperature pressure and density, respectively while **(D)** represents the radius of gyration of the complex for the time duration of 10 nanoseconds

Supplementary Table 1: IFN-γ epitope prediction for the top HTL epitopes

Serial No.	Epitope Name	Sequence	Method	Result	Score
1	Epitope_1	VRQEAIARALARAAA	MERCI	POSITIVE	4
2	Epitope_2	QVRQEAIARALARAA	MERCI	POSITIVE	3
3	Epitope_3	DQVRQEAIARALARA	MERCI	POSITIVE	3
4	Epitope_4	RDQVRQEAIARALAR	MERCI	POSITIVE	5
5	Epitope_5	YRDQVRQEAIARALA	MERCI	POSITIVE	7
6	Epitope_6	KYYAEMQTTLATVDK	MERCI	POSITIVE	1
7	Epitope_7	FLAHLLVQASQPWKA	MERCI	POSITIVE	1
8	Epitope_8	PKYYAEMQTTLATVD	SVM	POSITIVE	0.4
9	Epitope_9	QELRAQIAQQRIAQR	SVM	POSITIVE	0.5
10	Epitope_10	IQELRAQIAQQRIAQ	SVM	POSITIVE	0.5
11	Epitope_11	YYAEMQTTLATVDKA	MERCI	POSITIVE	1
12	Epitope_12	LAHLLVQASQPWKAL	MERCI	POSITIVE	3
13	Epitope_13	YAEMQTTLATVDKAK	MERCI	POSITIVE	1
14	Epitope_14	QYRDQVRQEAIARAL	MERCI	POSITIVE	6
